# Effect of the methanolic extracts of different parts of *Ferula assa-foetida* on naloxone-induced withdrawal behavior in morphine-dependent mice

**Published:** 2017

**Authors:** Mahnaz Khanavi, Sajad Maadani, Behnaz Farahanikia, Mahdieh Eftekhari, Mohammad Sharifzadeh

**Affiliations:** 1 Department of Pharmacognosy, Faculty of Pharmacy and Persian Medicine and Pharmacy Research Center, Tehran University of Medical Sciences, Tehran, Iran; 2 Faculty of Land and Food Systems, University of British Columbia, Vancouver, BC, Canada; 3 Department of Toxicology and Pharmacology and Pharmaceutical Sciences Research Center, Tehran University of Medical Sciences, Tehran, Iran

**Keywords:** Ferula assa-foetida, Extract, Gamma-aminobutyric acid (GABA), Morphine dependence, Withdrawal syndrome

## Abstract

**Objective:**

*Ferula assa-foetida,* a native species in Iran, is used for treatment of several diseases particularly for neurological disorders in Iranian Traditional Medicine. The aim of this study is to investigate the effect of methanolic roots, fruits, and aerial parts extracts of *Ferula assa-foetida* on withdrawal syndrome in morphine-dependent mice.

**Materials and Methods:**

Aerial parts, roots, and fruits of the plant were separately extracted with 80% MeOH. For induction of dependence, morphine (50, 50 and 75 mg/kg) was injected subcutaneously three times daily (10 am, 1 pm and 4 pm) for three days and a last dose of morphine (50 mg/kg) was administrated on the fourth day. Withdrawal syndrome was induced by injection of naloxone (5 mg/kg, intraperitoneal) 2 hr after the final dose of morphine. Different doses of the extracts were administered i.p. 60 minutes before naloxone injection and withdrawal sign was recorded 2 minutes after naloxone injection for a period of 60 minutes.

**Results:**

Pre-treatment of animals with different doses (2.5, 5, 10, 20 mg/kg) of methanolic extract of the aerial parts of *F. assa-foetida* caused a significant decrease in naloxone-induced behavior. Intraperitoneal administration of different doses (10, 15, 20, 25 mg/kg) of methanolic extract of the fruit significantly reduced the naloxone-induced withdrawal behavior (p<0.001).

**Conclusion:**

It might be concluded that the extracts of *Ferula assa-foetida* affect morphine withdrawal syndrome possibly via interference with the neurotransmitters in nervous system.

## Introduction

*Ferula assa-foetida* (Apiaceae) is a Persian native species of *Ferula* genus with a significant background in traditional medicine of different countries. The plant has been used in the treatment of various diseases such as gastro-intestinal disorders, asthma, epilepsy and other ailments with neurological origin (Iranshahy and Iranshahi, 2011 ▶). Interestingly, Nepali people use assafoetida as a sedative agent whereas Americans people use it as a stimulant agent (Eigner and Scholz, 1999 ▶). There are several reports on the biological effects of *F. assa-foetida* as an antispasmodic (Fatehi et al., 2004 ▶), analgesic, anti-inflammatory (Bagheri et al., 2014 ▶), sedative (Gholamnezhad et al., 2012 ▶), nervine, anthelmintic and antioxidant agent (Khajeh et al., 2005 ▶). According to previous studies, some *Ferula* species such as* F. persica* and* F. gummosa *reduce the signs of morphine-withdrawal syndrome in mice (Jadidi et al., 2011 ▶; Ramezani et al., 2001 ▶)

This herb contains various active ingredients including ferulic acid (Lee et al., 2009 ▶), sulfur-containing compounds with disulfide structure as major components (Samadi et al., 2016 ▶), coumarin derivatives e.g. umbelliferone (Bandyopadhyay et al., 2006 ▶), α-pinene, β-pinene, thymol, carvacrol, and 2-borneol (Bamoniri and Mazoochi, 2009 ▶).

Based on studies on morphine dependency, many different factors play roles in the induction of morphine withdrawal syndrome such as noradrenergic system (Ambrosio et al., 1997 ▶), serotonergic system (Mohajel Naebi and Asadi, 2009 ▶), dopaminergic system (Chartoff et al., 2009 ▶), adenosine receptor agonists (Bailey et al., 2004 ▶), and protein kinase inhibitors (Gabra et al., 2008 ▶).

According to the pharmacological studies on *F. assa-foetida* and its ingredients, this plant has a significant effect on the nervous system and neurotransmitters' pathways and could be useful for treatment of morphine withdrawal behavior. For example, ferulic acid inhibits neuronal and inducible nitric oxide synthase and significantly enhances expression of gamma***-***amino butyric acid (GABA_B1)_ receptor in cerebral ischemia in rats (Cheng et al., 2010 ▶). β-pinene, as a major compound of *F. assa-foetida *essential oil, exerts supraspinal antinociceptive action in rats (Liapi et al., 2007 ▶). Borneol, the other compound found in the plant oil, produces a highly efficacious positive modulation of GABA_A_ receptors (Granger et al., 2005 ▶). Also, carvacrol has shown antidepressant effects in the tail suspension and forced swimming tests (Melo et al., 2011 ▶). However, there is no sufficient evidence to prove various effects and mechanisms of action of *F. assa-foetida* on the nervous system.

In this study, we investigated the effects of the extract of roots, fruits and aerial parts of *F. assa-foetida* on naloxone-induced withdrawal behavior (jumping, grooming, rearing, wet dog shake, stool weight, and weight loss) in morphine-dependent mice with consideration of its possible use in the management of morphine withdrawal syndrome. 

## Materials and Methods

**Plant material**

Aerial parts, roots, and fruits of the plant were collected from Neyshabur, province of Khorasan, Iran in April 2010. After identification, a voucher specimen (TEH-6706) was deposited at the Herbarium of the Faculty of Pharmacy, Tehran University of Medical Sciences, Tehran, Iran.

**Preparation of extracts**

For this purpose, 100 g of dried and finely powdered roots, aerial parts, and fruits (100 g each) were separately extracted with 80% methanol (MeOH) at room temperature for 72 hr. This procedure was repeated 3 times. The MeOH extracts were concentrated under reduced pressure. Each extract was then washed with 100 ml hexane for three times. The roots, aerial parts, and fruits extracts were separately stored at 4 ^◦^C in sealed vials until usage.

**Materials**

Morphine sulfate (Temad, Tehran, Iran), naloxone hydrochloride, methanol and hexane (Merck, Darmstedt, Germany) were used.

**Animals**

Male albino mice (20-30 g) were purchased from Pasture Institute of Iran, Tehran, Iran. Animals were housed under standard conditions of humidity and temperature (50 ± 5%, 25 ± 2°C) with 12 hr dark cycle (7 pm-7 am). Mice were divided into two groups, namely the experimental group that was treated with different doses of the samples (roots, fruits and aerial parts extracts) and control groups that received saline (5 ml/kg). Eight mice were used in each group. Each animal was used only once.

**Induction of morphine dependence**

According to other studies, for induction of morphine dependence, morphine was injected subcutaneously (s.c.) to mice at the doses of 50, 50, and 75 mg/kg three times daily (10 am, 1 pm, and 4 pm) for 3 days (Sharifzadeh et al., 2006; Zarrindast et al., 1995 ▶). The higher daily dose, injected at 4 pm, aimed to minimize any overnight withdrawal. On day 4, mice received a last dose of morphine (50 mg/kg at 10 am). Groups of mice, each containing eight animals, were chosen randomly for the experiment.

**Measurement of withdrawal syndrome**

Withdrawal syndrome was induced by intraperitoneal (i.p.) injection of naloxone (5 mg/kg), two hr after the last administration of morphine. Then, each animal was individually placed in a glass cylinder (28 cm diameter, 30 cm height) and withdrawal signs were recorded for 60 min.

**Extracts treatment**

Different doses of methanolic extracts of root (0.001, 0.005, 0.01, 0.05 mg/kg), fruits (2.5, 5, 10, 20 mg/kg) and aerial parts (10, 15, 20, 25 mg/kg) of *F. assa-foetida* were administered i.p. 60 min before naloxone injection and evaluation of withdrawal signs was started 2 min after naloxone injection for a period of 60 min. Doses were chosen and modified based on previous studies (Jadidi et al., 2011 ▶; Ramezani et al., 2001 ▶) and a primary screening of the extracts. The mean ± SEM of the naloxone-induced withdrawal signs was determined for eight mice. Saline was used as a control for all types of extracts.

**Statistical analyses**

Data was analyzed by one way ANOVA and by the Newman-keuls *post-hoc* test. Differences between means (mean ± SEM) were considered statistically significant if p<0.05.

## Results

**Effect** **s** **of the root extract**

**Figure 1:**
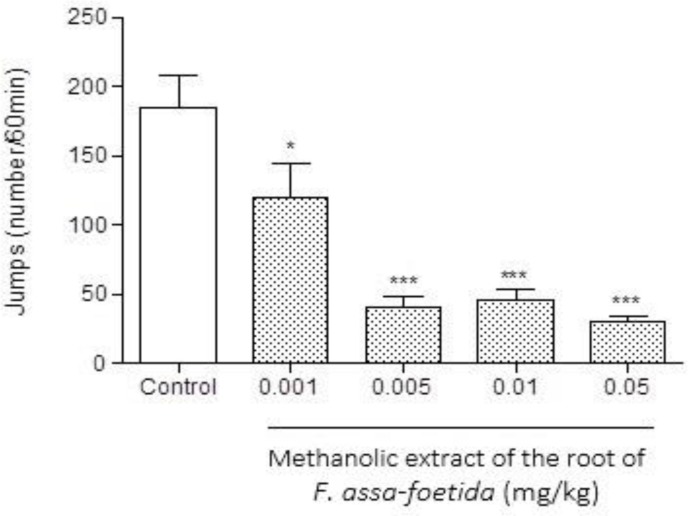
Effects of different doses of methanolic extract of the roots of *Ferula assa-foetida* on naloxone-induced jumping in morphine-dependent mice. Animals were treated subcutaneously with morphine three times/day (50, 50 and 75 mg/kg) for 3 days; the last dose of morphine (50 mg/kg) was injected on day 4, in order to develop dependence to morphine. Naloxone (5 mg/kg, i.p.) was injected 2 hr after administration of the last dose of morphine. Different doses of the methanolic extract were administrated one hour before naloxone injection. Control group received saline (5 ml/kg). The frequency of jumping was recorded for 60 min after naloxone injection. Each bar is the mean ± SEM of eight animals. *** p<0.001 and * p<0.05 show statistical difference from control group

**Figure 2:**
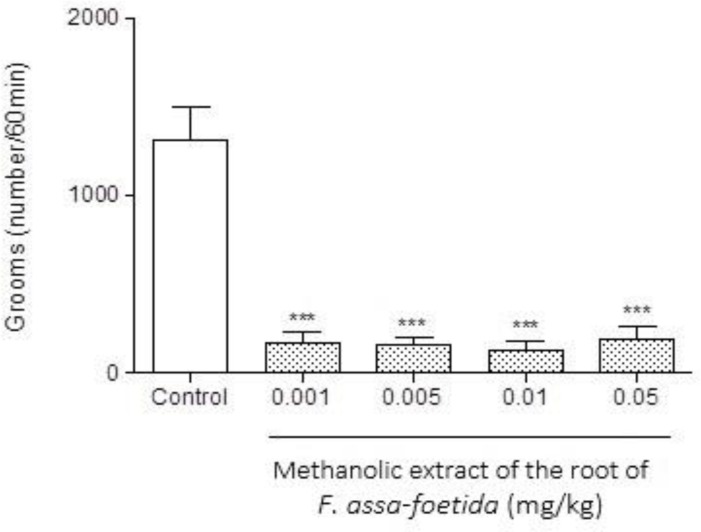
Effects of different doses of methanolic extract of the roots of *Ferula assa-foetida* on naloxone-induced grooming in morphine-dependent mice. Animals were treated subcutaneously with morphine three times/daily (50, 50 and 75 mg/kg) for 3 days; the last dose of morphine (50 mg/kg) was injected on day 4, in order to develop dependence to morphine. Naloxone (5 mg/kg, i.p.) was injected 2 hr after administration of the last dose of morphine. Different doses of the methanolic extract were administrated one hour before naloxone injection. Control group received saline (5 ml/kg). The frequency of grooming was recorded for 60 min after naloxone injection. Each bar is the mean ± SEM of eight animals. *** p<0.001 shows statistical difference from control group

**Figure 3:**
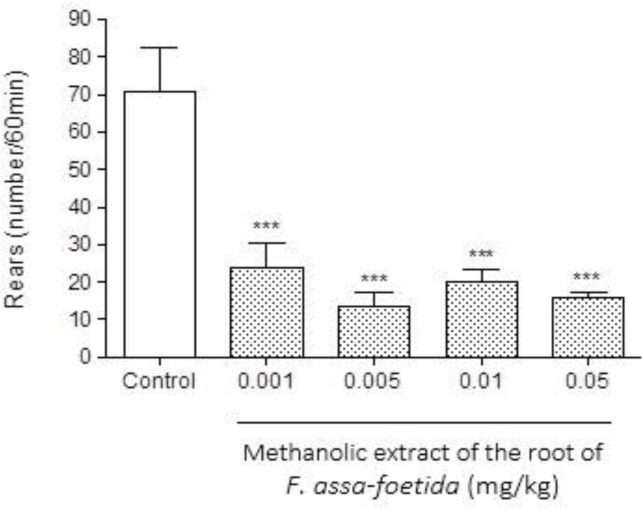
Effects of different doses of methanolic extract of the roots of *Ferula assa-foetida* on naloxone-induced rearing in morphine-dependent mice. Animals were treated subcutaneously with morphine three times/day (50, 50 and 75 mg/kg) for 3 days; the last dose of morphine (50 mg/kg) was injected on day 4, in order to develop dependence to morphine. Naloxone (5 mg/kg, i.p.) was injected 2 hr after administration of the last dose of morphine. Different doses of the methanolic extract were administrated one hour before naloxone injection. Control group received saline (5 ml/kg). The frequency of rearing was recorded for 60 min after naloxone injection. Each bar is the mean ± SEM of eight animals. *** p<0.001 show statistical difference from control group

Intraperitoneal injections of different doses (0.001, 0.005, 0.01, 0.05 mg/kg) of methanolic extract of *F. assa-foetida* roots 60 min before naloxone (5 mg/kg, i.p.) administration showed a significant decrease in naloxone-induced withdrawal behavior in morphine-dependent animals compared to saline (p<0.001 ) (Figures 1-3 and Table 1). The dose of 0.05 mg/kg, demonstrated a remarkable decrease in jumping (Figure 1), and administration of the extract at the dose of 0.01 mg/kg showed a significant decrease in grooming (Figure 2), wet dog shake (Table 1), and weight loss (Table 1) (p<0.001).

**Effect** **s** **of aerial parts extract**

**Figure 4:**
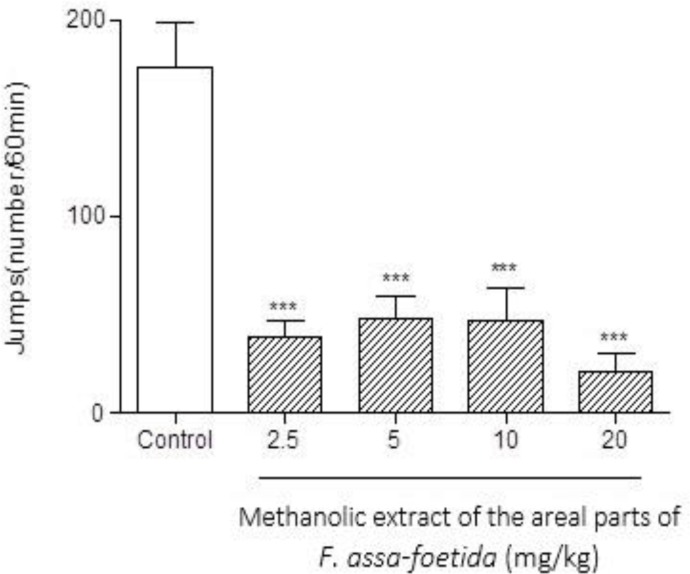
Effects of different doses of methanolic extract of the aerial parts of *Ferula assa-foetida* on naloxone-induced jumping in morphine-dependent mice. Animals were treated subcutaneously with morphine three times/day (50, 50 and 75 mg/kg) for 3 days; the last dose of morphine (50 mg/kg) was injected on day 4, in order to develop dependence to morphine. Naloxone (5 mg/kg, i.p.) was injected 2 hr after administration of the last dose of morphine. Different doses of the methanolic extract were administrated one hour before naloxone injection. Control group received saline (5 ml/kg). The frequency of jumping was recorded for 60 min after naloxone injection. Each bar is the mean ± SEM of eight animals. *** p<0.001 shows statistical difference from control group

**Figure 5:**
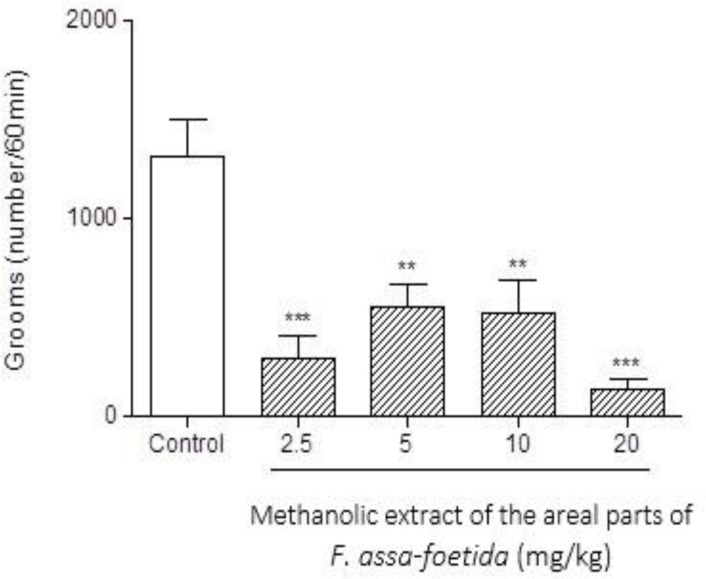
Effects of different doses of methanolic extract of the aerial parts of *Ferula assa-foetida* on naloxone-induced grooming in morphine-dependent mice. Animals were treated subcutaneously with morphine three times/day (50, 50 and 75 mg/kg) for 3 days; the last dose of morphine (50 mg/kg) was injected on day 4, in order to develop dependence to morphine. Naloxone (5 mg/kg, i.p.) was injected 2 hr after administration of the last dose of morphine. Different doses of the methanolic extract were administrated one hour before naloxone injection. Control group received saline (5 ml/kg). The frequency of grooming was recorded during 60 minutes after naloxane injection. Each bar is the mean ± SEM of eight animals. *** p<0.001 and **p<0.01 show statistical difference from control group

**Figure 6:**
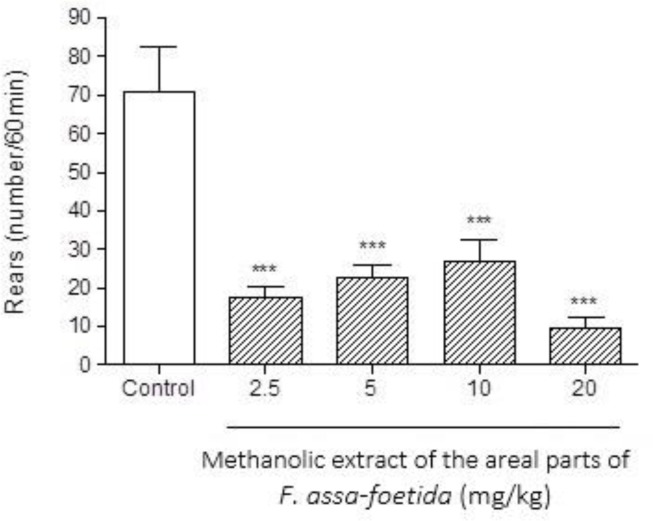
Effects of different doses of methanolic extract of the aerial parts of *Ferula assa-foetida* on naloxone-induced rearing in morphine-dependent mice. Animals were treated subcutaneously with morphine three times/day (50, 50 and 75 mg/kg) for 3 days; the last dose of morphine (50 mg/kg) was injected on day 4, in order to develop dependence to morphine. Naloxone (5 mg/kg, i.p.) was injected 2 hr after administration of the last dose of morphine. Different doses of the methanolic extract were administrated one hour before naloxone injection. Control group received saline (5 ml/kg). The frequency of rearing was recorded during 60 min after naloxone injection. Each bar is the mean ± SEM of eight animals. *** p<0.001 shows statistical difference from control group

Intraperitoneal injections of different doses (2.5, 5, 10, 20 mg/kg) of methanolic extract of the aerial parts of *F. assa-foetida* 60 min before naloxone (5 mg/kg i.p.) administration showed a significant decrease in naloxone-induced behavior in morphine-dependent animals. The highest administered dose, 20 mg/kg, showed a remarkable decrease in almost all measured parameters (p<0.001). (Figures 4-6 and Table 1).

**Effect of fruit extract**

**Figure 7:**
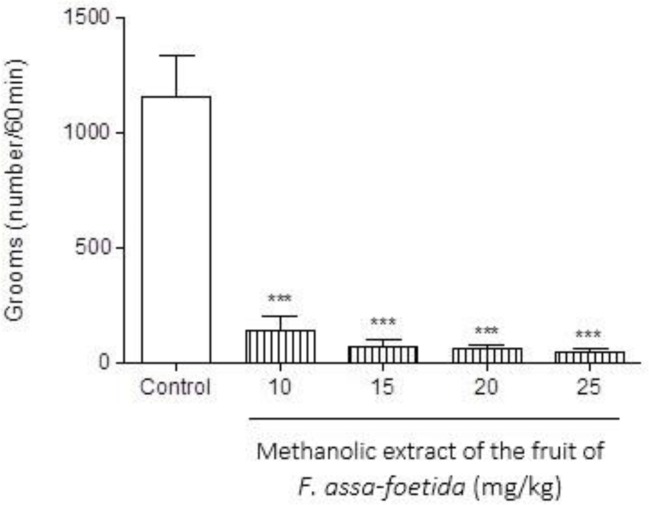
Effects of different doses of methanolic extract of the fruits of *Ferula assa-foetida* on naloxone-induced jumping in morphine-dependent mice. Animals were treated subcutaneously with morphine three times/day (50, 50 and 75 mg/kg) for 3 days; the last dose of morphine (50 mg/kg) was injected on day 4, in order to develop dependence to morphine. Naloxone (5 mg/kg, i.p.) was injected 2 hr after administration of the last dose of morphine. Different doses of the methanolic extract were administrated one hour before naloxone injection. Control group received saline (5 ml/kg). The frequency of jumping was recorded for 60 min after naloxone injection. Each bar is the mean ± SEM of eight animals. *** p<0.001 shows statistical difference from control group

**Figure 8:**
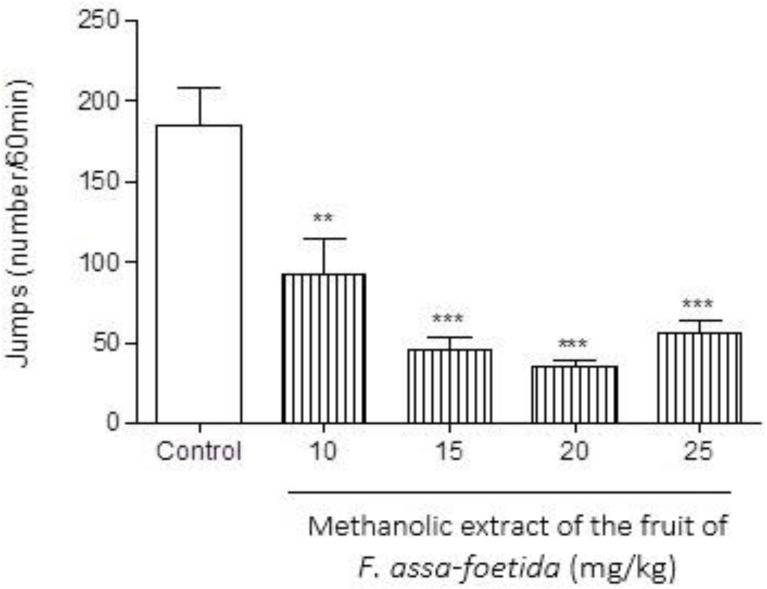
Effects of different doses of methanolic extract of the fruits of *Ferula assa-foetida* on naloxone-induced grooming in morphine-dependent mice. Animals were treated subcutaneously with morphine three times/day (50, 50 and 75 mg/kg) for 3 days; the last dose of morphine (50 mg/kg) was injected on day 4, in order to develop dependence to morphine. Naloxone (5 mg/kg,i.p.) was injected 2 hr after administration of the last dose of morphine. Different doses of the methanolic extract were administrated one hour before naloxone injection. Control group received saline (5 ml/kg). The frequency of grooming was recorded for 60 min after naloxone injection. Each bar is the mean ± SEM of eight animals. *** p<0.001 shows statistical difference from control group

**Figure 9:**
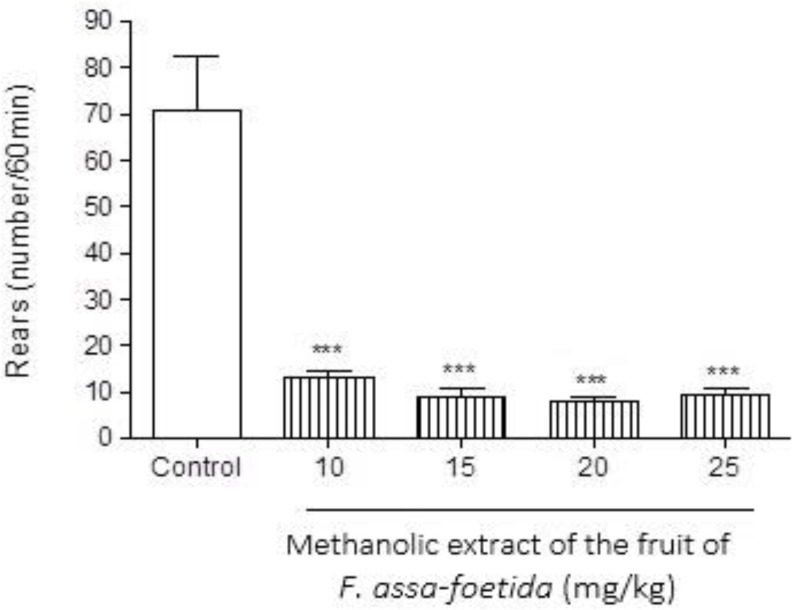
Effects of different doses of methanolic extract of the fruits of *Ferula assa-foetida* on naloxone-induced rearing in morphine-dependent mice. Animals were treated subcutaneously with morphine three times/day (50, 50 and 75 mg/kg) for 3 days; the last dose of morphine (50 mg/kg) was injected on day 4, in order to develop dependence to morphine. Naloxone (5 mg/kg, i.p.) was injected 2 hr after administration of the last dose of morphine. Different doses of the methanolic extract were administrated one hour before naloxone injection. Control group received saline (5 ml/kg). The frequency of rearing was recorded for 60 min after naloxone injection. Each bar is the mean ± SEM of eight animals. *** p<0.001 shows statistical difference from control group

Pre-treatment of animals with different doses (10, 15, 20, 25 mg/kg) of methanolic extract of *F. assa-foetida* fruits 60 min before naloxone (5 mg/kg i.p.) administration showed a significant decrease in naloxone-induced behavior in morphine-dependent animals (Figures 7-9). The dose of 20 mg/kg displayed a notable decrease in jumping (Figure 7), and wet dog shake (Table 1) (p<0.001).

**Table 1:** Effect of different doses of methanolic extract of the roots (A), aerial parts (B) and fruits (C) of *Ferula assa-foetida* on naloxone-induced wet dog shake, stool weight and weight loss in morphine-dependent mice

	**Treatment (mg/kg) **	**Wet dog shake** **(Number/min)**	**Stool weight** ** (g)**	**Weight loss** ** (g)**	
**A **	Control	17.70 ± 2.60	0.36 ± 0.02	1.70 ± 0.10	
	0.001	2.50 ± 0.5o***	0.10 ± 0.01***	0.70 ± 0.10***	
	0.005	4.25 ± 1.00***	0.09 ± 0.01***	0.40 ± 0.08***	
	0.01	1.62 ±0.53***	0.20 ± 0.03***	0.40 ± 0.07***	
	0.05	2.11 ±0.35***	0.20 ± 0.01***	0.70 ± 0.10***	
**B**	Control	17.70 ± 2.60	0.36 ± 0.02	1.70 ± 0.10	
	2.5	3.30 ± 0.50***	0.10 ±0.01***	0.60 ± 0.09***	
	5	4.20 ± 0.60***	0.10 ± 0.01***	0.50 ± 0.09***	
	10	7.00 ± 1.80***	0.10 ± 0.01***	0.50 ± 0.06***	
	20	4.00 ± 1.40***	0.23 ± 0.04***	0.40 ± 0.06***	
**C**	Control	17.70 ± 2.60	0.36 ± 0.02	1.70 ± 0.11	
	10	2.30 ± 0.56***	0.13 ± 0.01**	0.30 ± 0.50***	
	15	3.20 ± 0.80***	0.08 ±0.01***	0.48 ± 0.14***	
	20	1.75 ±0.48***	0.21 ±0.01***	0.40 ± 0.06***	
	25	3.00 ± 0.58***	0.15 ± 0.02***	0.41 ± 0.08***

The signs were recorded for 60 minutes after naloxone injection. Data is represented as the mean ± SEM of eight animals.

** p<0.01 and

*** p<0.001 show statistical difference from control group.

Result of the present study demonstrated that the methanolic extracts of roots, fruits and aerial parts of *F. assa-foetida* show significant decrease in naloxone-induced withdrawal behavior in morphine-dependent mice even at their lowest dose. The root extract displayed stronger inhibitory effect on naloxone-induced withdrawal behavior in comparison to the aerial parts and fruits extracts since it was effective at lower doses (0.005 mg/kg). 

## Discussion

Several studies have mentioned the major active ingredients of *F. assa-foetida* such as ferulic acid, carvacrol, thymol, etc. and their essential role in the nervous system and neurotransmitters' pathways (Calabrese et al., 2007 ▶; Waliwitiya et al., 2010 ▶; Zotti et al., 2013 ▶). Acute and chronic morphine treatment produced an increase in Ca^2+^-dependent nitric oxide synthase (NOS) in mice brain. The three isoforms of nitric oxide synthase which form endogenous nitric oxide are neuronal, endothelial, and inducible nitric oxide synthase. It seems that inducible NOS (iNOS) and neuronal NOS (nNOS) are involved in morphine dependence or withdrawal syndrome. Central signs of morphine dependence may be associated with nitric oxide produced by nNOS. Nitric oxide synthase inhibitors (nNOS or iNOS inhibitors) contribute to treatment of opioid dependence or tolerance and its withdrawal syndrome (Cao et al., 2006 ▶; Toda et al., 2009 ▶)

*F. assa-foetida* and its constituent ferulic acid, inhibit neuronal (nNOS) and inducible (iNOS) nitric oxide synthase (Koh, 2012 ▶). Also, a new caffeic acid cinnamyl ester isolated from *F. assa-foetida* inhibits LPS-induced nitric oxide production (Song et al., 2008 ▶). 

Moreover, mitogen-activated protein kinase MAPK, is activated during morphine withdrawal syndrome in the locus coeruleus and directly affects μ opioid receptor (Schulz and Höllt, 1998 ▶). There are some other known members of MAPK family including extracellular signal-regulated kinase (ERK), c-jun N-terminal kinase (JNK), and p38 MAPK, and ferulic acid can inhibit p38 (MAPK) phosphorylation (Abd El‐Razek et al., 2007 ▶; Cheng et al., 2010 ▶) Long-term morphine exposure induces the activation of this family in the central and peripheral nervous system as well. Application of a MAPK inhibitor can reduce morphine tolerance and dependence (Cheng et al., 2010 ▶). Additionally, increase in phospho-ERK1/2 (pERK1/2) expression during morphine withdrawal syndrome can be reduced by pretreatment with non-selective NOS inhibitor, nNOS inhibitor, or iNOS inhibitor, in rats spinal cord (Cao et al., 2006 ▶). Thus, it seems that *F. assa-foetida* extracts can cause inhibitory effect on signs of morphine withdrawal syndrome through inhibition of nitric oxide synthase and decreasing NO production, as well as inhibition of MAPK phosphorylation.

Ferulic acid and galbanic acid can inhibit p38 MAPK phosphorylation, and it has been recently reported that a p38 MAPK inhibitor could be regarded as a treatment for thermal hyperalgesia induced by morphine withdrawal (Bederson et al., 1990 ▶; Sung et al., 2005 ▶)

Moreover, ferulic acid enhances the expression of gamma-aminobutyric acid type B receptor subunit 1 (GABA_B1_) (Cheng et al., 2010 ▶). Moreover, α-pinene has a positive modulating action at GABA_A_ receptors (Aoshima and Hamamoto, 1999 ▶). Borneol, another main compound of the plant, produces a highly efficacious positive modulation of GABA_A_ receptors (Granger et al., 2005 ▶); furthermore, borneol and carvacrol showed anticonvulsant effect against PTZ-induced convulsions and maximal electroshock (MES). These effects are probably mediated through modulation of GABAergic system by enhancement of GABA_A_-BZD receptor (Quintans-Júnior et al., 2010 ▶).

Recent studies have reported that thymol is a positive allosteric modulator of the GABA_A_ receptor and enhances its activity (García et al., 2006 ▶).

Several reports have shown that morphine causes an increase in whole brain GABA concentration in mice (Zarrindast and Mousa-Ahmadi, 1999 ▶) and also increases GABA in discrete parts of the thalamus and spinal cord of rats (Kuriyama and Yoneda, 1978 ▶). It has been suggested that GABA-ergic and opiopeptidergic systems are interconnected through μ-opioid receptors (Desarmenien et al., 1984 ▶). Both GABA_A_ and GABA_B_ receptor subtypes may have an inhibitory influence on naloxone-induced withdrawal signs such as jumping. Activation of GABA_B_ receptor in the LC reduces precipitated morphine withdrawal symptom (Riahi et al., 2009 ▶; Zarrindast and Mousa-Ahmadi, 1999 ▶).

Beta-pinene exerted supraspinal anti-nociceptive actions in rats only and reversed the antinociceptive effect of morphine which was comparable to naloxone; probably beta-pinene acts as a partial agonist for the μ-opioid receptors. From structure-activity relationships of the pair naloxone + beta -pinene, it was shown that similarities exist in the stereochemistry and respective atomic charges of these molecules (Liapi et al., 2007 ▶).

Carvacrol presents antidepressant effects in the forced swimming and tail suspension tests. This effect seems to be dependent on its interaction with the dopaminergic system, but not with the serotonergic and noradrenergic systems (Melo et al., 2011 ▶). It could decrease the number of grooming in the open-field test (Melo et al., 2010 ▶). 

Other species of this genus such as *Ferula gummosa* Boiss. and *Ferula persica* can also reduce the signs of morphine withdrawal syndrome in mice and this effect may be related in part to the presence of terpenoid compounds (Jadidi et al., 2011 ▶; Ramezani et al., 2001 ▶). As well, the results of our study on *F. assa-foetida* showed a significant decrease in naloxone-induced withdrawal behavior in morphine-dependent mice even at the lowest dose.

In conclusion, our study demonstrated that the extracts of the roots, fruits and the aerial parts of *F. assa-foetida* decreased naloxone-induced withdrawal signs in morphine-dependent animals via a possible interaction with the inhibitory neurotransmitter system. Further research is needed to isolate the active components and to reveal the exact effect(s) of various components of *F. assa-foetida* L. extracts on morphine withdrawal syndrome.
